# Controlling bubble generation by femtosecond laser-induced filamentation

**DOI:** 10.1038/s41598-022-20066-1

**Published:** 2022-09-21

**Authors:** D. Chaitanya Kumar Rao, Veena S. Mooss, Yogeshwar Nath Mishra, Dag Hanstorp

**Affiliations:** 1 Department of Physics, University of Gothenburg, 41296 Gothenburg, Sweden; 2 NASA-Jet Propulsion Laboratory, California Institute of Technology, Pasadena, CA 91109 USA; 3 Present Address: Department of Aerospace Engineering, Indian Institute of Technology Kanpur, Kanpur, 208016 India

**Keywords:** Mechanical engineering, Fluid dynamics, Applied optics, Biomedical engineering

## Abstract

Femtosecond laser-induced optical breakdown in liquids results in filamentation, which involves the formation and collapse of bubbles. In the present work, we elucidate spatio-temporal evolution, interaction, and dynamics of the filamentation-induced bubbles in a liquid pool as a function of a broad spectrum of laser pulse energies (∼1 to 800 µJ), liquid media (water, ethanol, and glycerol), and the number of laser pulses. Filament attributes such as length and diameter have been demarcated and accurately measured by employing multiple laser pulses and were observed to have a logarithmic dependence on laser energy, irrespective of the medium. The size distribution of persisting microbubbles is controlled by varying the pulse energy and the number of pulses. Our experimental results reveal that introducing consecutive pulses leads to strong interaction and coalescence of the pulsating bubbles via Bjerknes force due to laser-induced acoustic field generation. The successive pulses also influence the population density and size distribution of the micro-bubbles. We also explore the size, shape, and agglomeration of bubbles near the focal region by controlling the laser energy for different liquids. The insights from this work on filamentation-induced bubble dynamics can be of importance in diverse applications such as surface cleaning, fluid mixing and emulsification, and biomedical engineering.

## Introduction

Microbubble generation has applications in several fields ranging from medical diagnostics and pharmacology to water purification and food industry^1^. For instance, microbubbles are considered one of the most effective contrast agents in clinical ultrasound imaging^2^. Their high compressibility allows them to scatter ultrasound more effectively than red blood cells. Microbubbles have demonstrated great potential and extensive use in therapeutic applications such as targeted drug delivery, gene therapy, ultrasound surgery, tumor ablation^3–7^ and the removal of waste nanoparticles from the bloodstream^8^. Microbubbles have also been used for biofilm removal^9^, degradation of harmful compounds, water disinfection and cleaning/de-fouling of solid surfaces^10^. Therefore, it is important to investigate the controlled generation and manipulation of stable microbubbles.

The applications mentioned above have significantly augmented the need for robust bubble generation approaches that present a high degree of control over bubble characteristics such as mean bubble diameter, bubbling frequency, and polydispersity index (PDI). For instance, in the case of therapeutic and diagnostic applications, approximately one million well-defined microbubbles (PDI < 5%) and a mean diameter of the order of 10 μm are required^11^. Commercial microbubble generation commonly involves ultrasonication and mechanical agitation methods, which produce a significant amount of small microbubbles^12^. In these techniques, the generated bubbles predominantly result in polydisperse size distributions^13,14^. Although the commercial bubble generation approaches are known for their simplicity and robustness, they lack the precision in size, location and low PDI of microbubbles required for specialized laboratories and technological applications.

Optical cavitation was observed in liquids for the first time using Q-switched ruby lasers by Askar'yan et al.^15^ and was subsequently confirmed by Brewer et al.^16^. Carome et al.^17^ studied the formation of a single bubble with its shockwave obtained in a dust-free sample of distilled water. This study was extended and confirmed later by Lauterborn^18,19^, with the observation of the shock wave emitted upon breakdown via the compression of a small bubble. It has been established that ultra-short pulses (femtosecond pulse duration) lead to well-controlled cavitation compared to longer laser pulses (nanosecond and picosecond pulse duration). It was shown by Glezer et al.^20^ that femtosecond laser-induced breakdown (LIB) in water occurs at a very stable and low-energy threshold as compared with picosecond or nanosecond LIB, which produce unstable breakdown at energies near the threshold. Moreover, in the case of longer pulses, relatively large pulse energies (in the millijoule range) are required to achieve optical breakdown, resulting in bubble formation that exhibits stochastic behaviour. The considerable threshold energy also leads to uncontrolled tissue effects in surgical procedures^21^. In addition, Juhasz et al.^22^ have studied that the spatial range of shock waves induced by femtosecond laser pulses is considerably smaller than that of longer pulses. The reduced shock wave and micro-bubble effects of the femtosecond laser lead to more a confined tissue damage. Therefore, a more localized surgical effect is expected from a femtosecond laser than from a laser with large pulse widths. This indicates a potential benefit of femtosecond laser technology to intraocular microsurgery^22,23^. Therefore, ultra-short laser pulses are desirable for minimally invasive and highly localized surgical procedures resulting in minimal collateral tissue damage. Furthermore, it has been shown that, when compared to UV lasers, near-infrared lasers are not absorbed (at least to the first order) in ocular media and can pass through transparent and limited thickness translucent material, thus affecting tissue only at the laser beam focus^21^.

Femtosecond filamentation-induced bubble generation allows precise control and prediction of the size, location, and polydispersity of the generated bubbles. Furthermore, it is a non-intrusive mode of micro-bubble generation in a very short timescale. In liquids, femtosecond laser-induced filamentation (FLIF) initiates from the dynamic balance between the self-focusing (optical Kerr effect) and defocusing effect by plasma formation^24^. The formation of bubbles through FLIF is a complex process which comprises the following events: initially, the high-power laser irradiation inside the liquid medium generates the free electrons via multiphoton ionization and tunnel ionization due to extremely high peak power at the focal volume (> 10^12^ W/cm^2^)^25,26^. This results in the rapid excitation, ionization, and dissociation of the liquid into plasma, having a high temperature (~ 10^4^ K) in the focal volume^27^. Thereafter, recombination processes commence, and the plasma is replaced by vaporized fluid mass, which constitutes micro-bubbles^28^. Depending on the laser energy, focusing conditions, and liquid medium, single (spherical or non-spherical) or multiple bubble formation may occur^29–31^. Laser pulse-induced bubbles are especially important as they assist in cellular microsurgery, removing thrombus (blood clot) from clogged arteries and clearing bile duct stones through breakup and lithotripsy^31–33^.

A number of studies have been conducted for generation of bubbles and acoustic signals in water^34–38^. Potemkin and Mareev^34^ observed the evolution of multiple cavitation bubbles in a single filament excited by a femtosecond laser pulse in water. Potemkin et al.^36^ further investigated different regimes of filamentation and corresponding dynamics of filament-induced shock waves and micro-bubbles in water using different focusing (including aberrations), laser parameters (pulse energy), and medium properties (linear absorption). Jukna et al.^37,38^ revealed the strong influence of pulse duration on the shape and intensity of the acoustic signals generated by filamentation of ultrashort terawatt laser pulses in water, which is related to the mechanism of superfilamention in water. Nevertheless, most studies on laser-assisted bubble dynamics are investigated on single bubbles, predominantly at low laser energies (< 200 µJ). Furthermore, the generation of laser-induced multiple bubbles is inadequately understood and is mainly subjected to theoretical works^39,40^. Thus, given the significance of cavitation bubbles in liquids, a great deal of research is required to comprehend the multiple bubble hydrodynamics during the laser-induced breakdown process. In addition, the interaction of micro-bubbles caused by temporally separated laser pulses is of great importance. For example, it can be pivotal to understand how the persisting cavitation bubble (or its fragments) of the previous pulse interacts with the bubbles generated by the new incoming pulse.

In the present work, we have presented a systematic and comprehensive experimental investigation of the microbubbles induced by temporally separated ultra-short pulses. An in-depth study of the influence of laser energy, number of pulses, and liquid medium on the bubble size, shape, and population density is explored in detail. Section “Experimental methodology” provides an overview of the experimental procedure. In section “Results and discussion”, detailed characteristics of filaments as a function of laser energy and pulses for different liquids are described. Section “Results and discussion” lays out the size distribution of micro-bubbles and their dissimilarity with varying laser energy and pulses. Section “Results and discussion” explains the dynamics of the single bubble near the focal region. The influence of laser energy on the bubble shape, size, and agglomeration for different liquids is elucidated in detail. Finally, in section “Results and discussion”, multi-pulse laser-induced bubble coalescence and the observation of bubble merging via Bjerknes force are revealed and characterized.

## Experimental methodology

**Figure 1:**
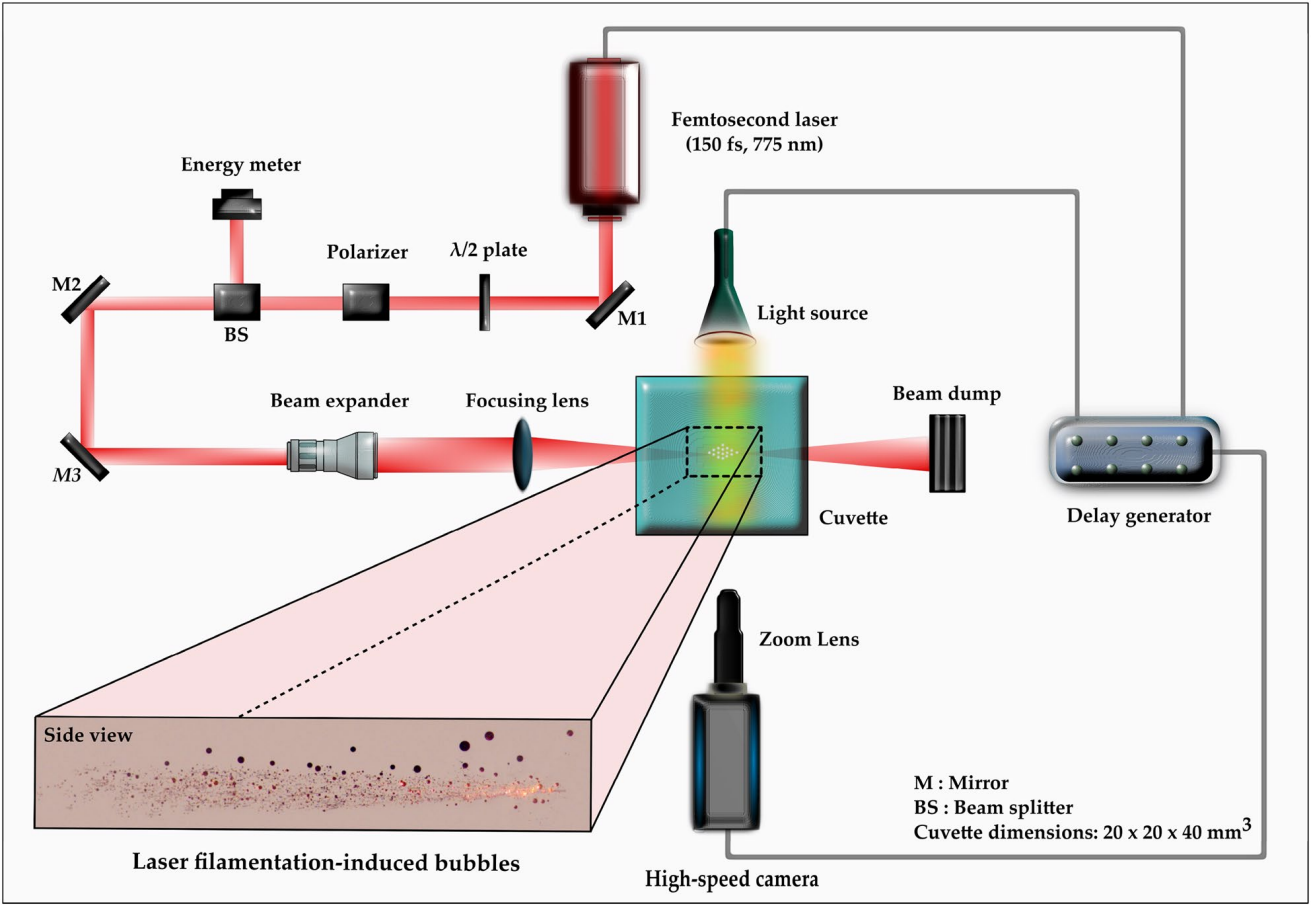
Schematic top-view of the experimental arrangement for micro-bubble creation and observation of bubble dynamics driven by femtosecond laser pulses. The figure was created with Inkscape, version 1.2 (https://inkscape.org).

The experimental arrangement for probing the dynamics of femtosecond laser-induced filamentation is illustrated in Fig. 1. Femtosecond laser pulses (pulse duration: 150 fs, central wavelength: 775 nm) were generated from a Ti–sapphire chirped-pulse amplification laser system (CPA 2001) at a repetition rate of 1 kHz. In the present work, we did not consider any pulse expansion in the water. Tsuji et al.^41^ measured the laser pulse duration after passing through a 20 mm length quartz cell containing water (similar to the current work) and showed negligible expansion of the laser pulse inside the water. The laser beam (diameter ~ 5 mm) was first expanded using a beam expander, which was subsequently focused into a quartz cuvette (20 mm × 20 mm × 40 mm) containing different liquids (de-ionized water, ethanol, and glycerol) by a plano-convex lens (50 mm focal length). The thickness of the quartz walls is ~ 1 mm, and its nonlinear refractive index (*n*_2_^*glass*^) is ≈ 4 × 10^–20^ m^2^/W, which is approximately similar to water (*n*_2_^*water*^ = 4.1 × 10^–20^ m^2^/W) and ethanol (*n*_2_^*ethanol*^ = 7.7 × 10^–20^ m^2^/W)^42^. Therefore, it can be presumed that the quartz wall does not alter the filamentation process. Moreover, adequate measures were taken to create the filaments deep inside the cuvette. The pulse energy was varied from 1 to 785 µJ. A half-wave plate and polarizing beam-splitter cube were utilized for laser power adjustment. A power meter was used to calibrate the laser pulse energy. The filament bundle was generated in the centre of the cuvette, which was visualized using a high-speed camera (Phantom Miro LAB310) and a zoom lens (Navitar 6.5X Zoom) at 3000 fps (exposure time of 330 µs). An LED light was used to illuminate the micro-bubbles. The spatial resolution of recorded images was 10.5 µm/pixel. To observe the dynamics of single bubble evolution, the frame rate of the high-speed camera was increased to 440,000 fps for ethanol (exposure time of 1.14 µs) and 630,000 fps for water (exposure time of 1.4 µs) at a spatial resolution of 13.7 µm/pixel. The images were processed and examined using an image analysis and processing program (Image-Pro Plus).

The high-speed camera, LED, and the femtosecond laser were synchronized via a delay generator (BNC 745 T). To achieve high repeatability, the experiments were performed at least 5 times.

## Results and discussion

### Global observations

**Figure 2:**
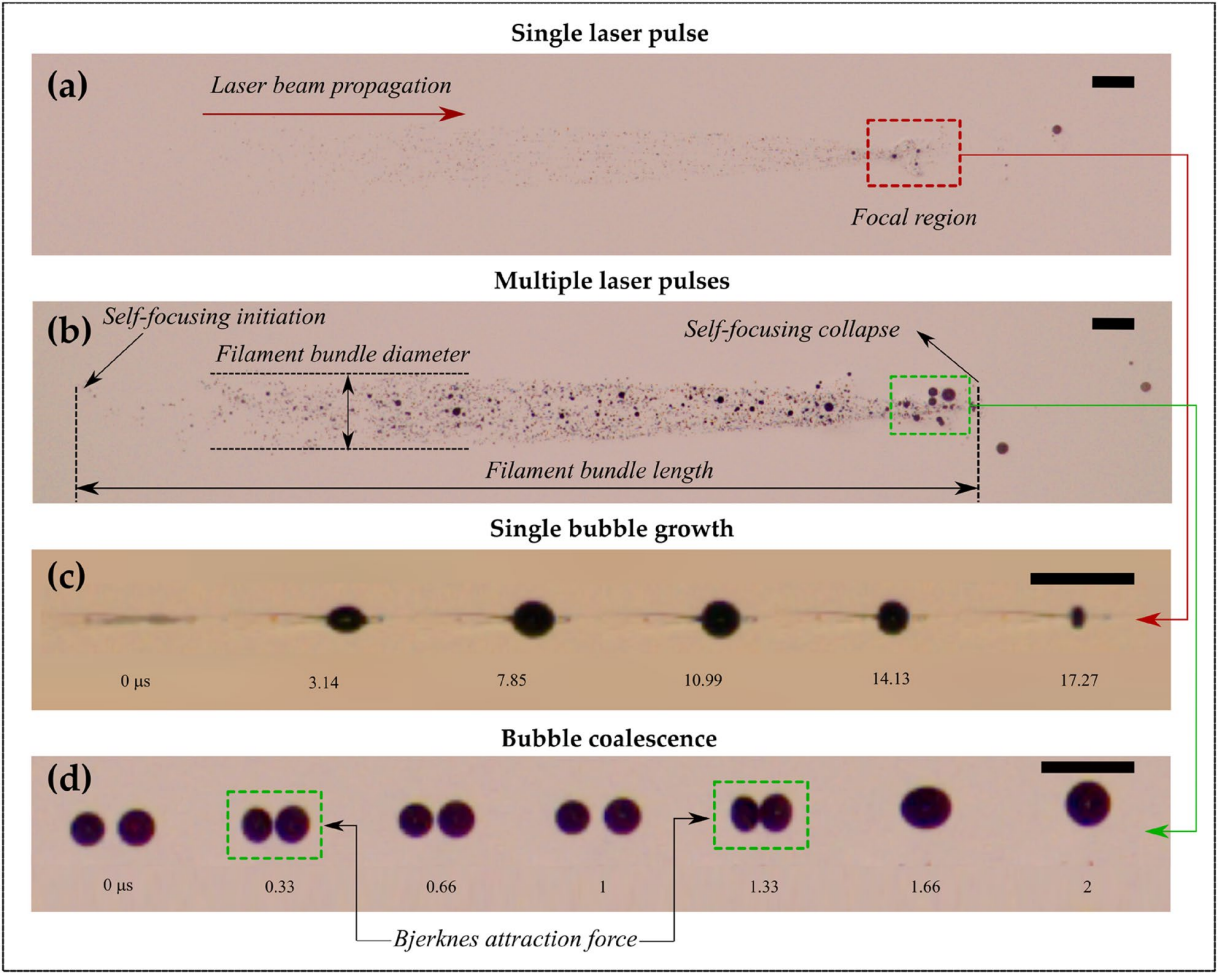
(**a**) Microbubble generation using a single femtosecond laser pulse-induced filamentation in ethanol; (**b**) Microbubble generation using multiple (30 pulses) femtosecond laser pulse-induced filamentation in ethanol at 1 kHz repetition rate; (**c**) Growth and collapse of a single bubble in the water near the geometrical focus (Δt = 0 µs represents the onset of bubble formation); and (**d**) Coalescence of micro-bubbles due to Bjerknes force (Δt = 0 µs represents the onset of bubble attraction). The sequence of events corresponds to the pulse energy of 785 µJ. The scale bar represents 500 µm.

When a high-power femtosecond laser pulse propagates through a liquid medium, a localized change in the refractive index ensues. This change in refractive index induces a lens-like effect that tends to focus the laser beam inside the medium (also known as self-focusing). When the laser power exceeds a critical threshold for self-focusing, the molecules in the irradiated area (reaching 10^13^–10^14^ W/cm^2^) undergo excitation and rapid ionization^43^. Since the irradiation intensity in the current work (> 10^13^ W/cm^2^) is sufficiently substantial to cause self-focusing, the pulse converges by itself, forming a filament or plasma channel. Eventually, multiple optical filaments converge in the focal region of the focusing lens resulting in a stream of microbubbles (see Fig. 2a,b) generated along the laser propagation axis where the direction of laser beam propagation is from left to right.

Figure 2 shows the global observations corresponding to the FLIF and associated processes. The bubbles seen in Fig. 2a,b are formed following the collapse of cavitating bubbles (see Fig. 2c), especially near the focal region. These persisting bubbles aid in the visualization of the filaments. The population density of persisting bubbles for a single pulse is low; therefore, it is rather difficult to accurately identify the length and diameter of the filament bundle. In the present work, it is observed that the total length of the filament bundle can be visualized more accurately when multiple pulses interact with the liquid medium. Accordingly, multiple laser pulses at 1 kHz repetition rate (up to 1000 pulses) are used to demarcate the filament characteristics (such as length and diameter).

Moreover, it is important to note that the filament length and diameter do not change when the number of laser pulses is increased. Figure 2a,b depict the images corresponding to ethanol upon introducing a single laser pulse and multiple pulses (30 pulses). Although the end of the filament is discernable for a single pulse, the onset of filamentation or starting point of the self-focus is unclear (Fig. 2a).

However, upon the introduction of multiple laser pulses, the starting point of the filament becomes more apparent when 30 consecutive laser pulses are introduced at 1 kHz repetition rate (Fig. 2b). This demonstrates that the interaction of multiple laser pulses with a liquid medium aids in accurately determining the filament length.

Near the focal region where the laser irradiation-induced electric field is at its maximum, laser-induced breakdown (LIB) occurs, resulting in the formation of a hot plasma. Therefore, a comparatively large single bubble grows and collapses near the focal region, as seen in Fig. 2c, where Δt = 0 µs represents the onset of bubble formation. The generated micro-bubbles are directed forward and backwards with average velocities of ~ 1 m/s in the proximity of the focal region and ~ 0.1 m/s away from the focal region (towards the self-focus position). The collapse of the microbubble imparts the fragments adequate momentum to disperse horizontally over distances of about 2 mm before decelerating and thereafter rising vertically in the direction of the liquid surface. Furthermore, it is found that when multiple laser pulses are introduced into the liquid medium, the bubbles strongly interact due to the Bjerknes attraction force (Fig. 2d). Here, Δt = 0 µs represents the onset of bubble attraction.

### Filament characteristics

Filamentation occurs when the input power (*P*_*in*_) exceeds the critical power (*P*_*cr*_), producing a continuous plasma channel. The critical power for self-focusing (*P*_*cr*_) is given by^44^.

1 $${P}_{cr}=\frac{{R}_{cr}{\lambda }^{2}}{8\pi {n}_{0}{n}_{2}}.$$

Here, *λ* is the wavelength of the laser, *n*_0_ and *n*_2_ are the linear and the nonlinear indices of refraction, which describe the intensity-dependent refractive index, *n* = *n*_0_ + *n*_2_*I*. For an axially symmetric collimated Gaussian beam, the nonlinearity parameter, *R*_*cr*_ = 3.77.

The *P*_*cr*_ for the liquids used in the present work, i.e., water, glycerol, and ethanol are 1.5, 1.2, and 0.9 MW, respectively. Multiple filaments are formed for most laser pulse energies due to the significantly higher input power than the self-focusing threshold. The present experimental observations are consistent with the study by Liu et al.^24^, in which multiple filaments were reported for a laser energy of ~ 1 µJ.

The number of filaments for particular laser energy can be estimated using^28^.

2 $$N\sim {P}_{in}/{P}_{fil}=\frac{{P}_{in}}{{{\left(\frac{\pi }{2}\right)}^{2}({\lambda }^{2}/2\pi n}_{0}{n}_{2})}.$$

**Figure 3:**
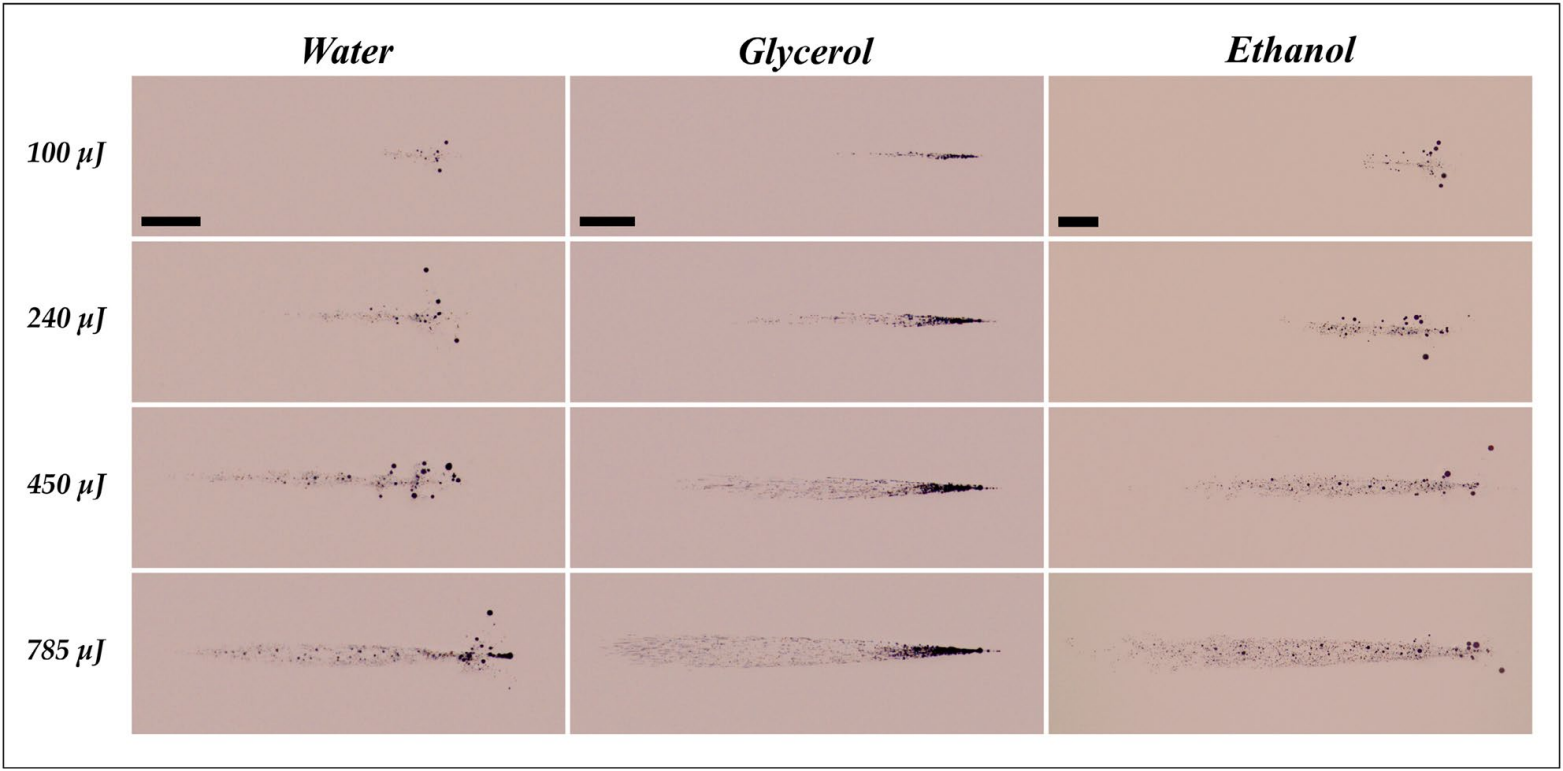
Femtosecond laser-induced filaments corresponding to water, glycerol, and ethanol at 100 µJ, 240 µJ, 450 µJ, and 785 µJ. The laser pulse comes from the left. The scale bar represents 1 mm.

Theoretically, the number of filaments for the lowest pulse energy used in the current work (1 µJ) for water, glycerol, and ethanol are approximately 2, 2, and 3, respectively. As the laser pulse energy increases, there is a consistent (linear) increment in the number of filaments. At the highest laser energy (785 µJ), the estimated number of filaments for water, glycerol, and ethanol are around 1400, 1800, and 2400, respectively. The higher number of filaments in the case of ethanol can be attributed to its lower bandgap energy (6 eV)^45^ compared to water (7.8 eV)^46^, which is also reflected in their respective filament length (see Fig. 3). It has been shown that the number of filaments in the air could also be governed by geometrical constraints and mutual interactions among filaments^47^. However, more evidence is needed to prove the effect of geometrical conditions and mutual interactions among filaments in the case of liquids.

Without any external focusing through a lens, a collimated laser beam having a Gaussian beam profile will self-focus at^44^.

3 $${Z}_{f}=\frac{0.367{ka}^{2}}{{\left\{{\left[{\left(\frac{{P}_{in}}{{P}_{cr}}\right)}^\frac{1}{2}- 0.852\right]}^{2}-0.0219\right\}}^\frac{1}{2} },$$

where *k* is the wavenumber in air, *a* is the radius at the 1/*e*^2^ level of the beam profile, and *ka*^2^ signifies the diffraction length. In the case of external focusing, using a lens of focal length *f*, the position of the self-focus will change to^44^.

4 $${Z}_{f}^{\mathrm{^{\prime}}}=\frac{{Z}_{f}f}{{Z}_{f} + f},$$

where ${Z}_{f}^{^{\prime}}$ is the distance between the focusing lens and the self-focus position. Equations 3 and 4 indicate that the starting point of the filament moves from the focal region towards the lens as the input laser power (${P}_{in}$) is raised above the critical power (*P*_*cr*_). This is also evident in the experimental observations and will be discussed in the forthcoming text.

**Figure 4:**
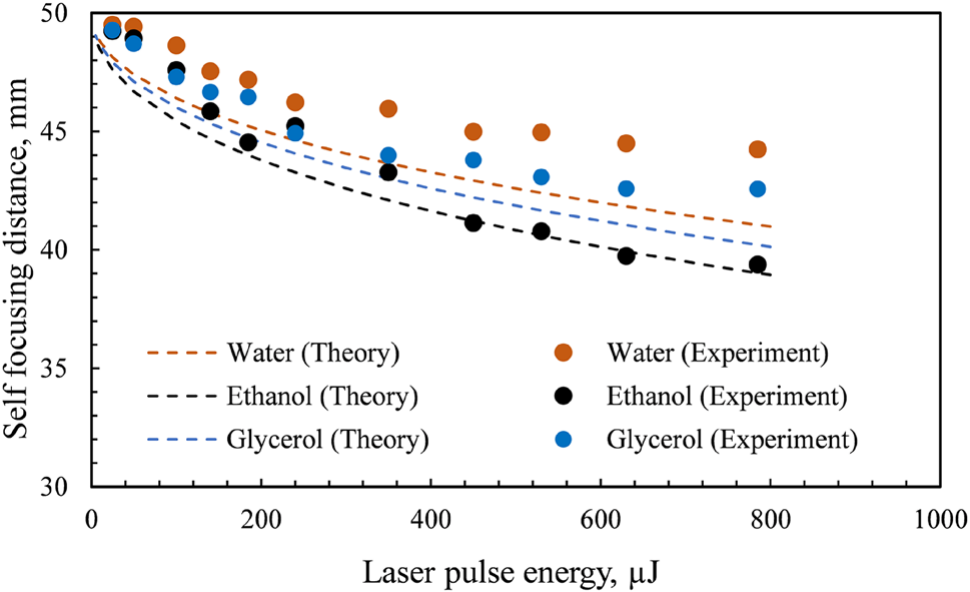
Experimental and theoretical variation of self-focusing distance with laser energy in different liquid media. The total error in the measurement of self-focusing distance is < 1%.

Figure 3 displays the representative images of filamentation in different liquid media at laser pulse energies varying from 100 to 785 µJ. It is apparent that the length and diameter of filament increase with an increase in the laser energy for all the liquids. Accordingly, the self-focusing distance (starting position of the filament) decreases with an increment in the laser energy. Self-focusing tends to occur closer to the focal region at low laser pulse energy (Fig. 4). In contrast, an increase in the laser energy decreases the self-focus distance, indicating that the self-focus position moves towards the focusing lens. The self-focusing distance can be indicated as the distance between the focusing lens and the self-focus position. It is observed that the self-focusing distance has a logarithmic dependence on the laser energy for all the liquids. Moreover, the experimental variation agrees well with Eq. 4.

The filament length can be expressed as the distance where the laser power is sufficiently high to ionize the liquid. The filament length at any pulse energy is maximum for ethanol, followed by glycerol and water. For instance, the filament length at the pulse energy of 785 µJ is maximum for ethanol (~ 10 mm), followed by glycerol (~ 7 mm) and then water (~ 5 mm). The variation in the filament length for different liquid media for the same laser energy can be ascribed to the difference in the bandgap energy of the medium^48^. For a medium with a low bandgap (6 eV for ethanol), the filament length is larger due to its higher tendency to ionize (i.e., high electron density) compared to a medium with relatively high bandgap energy (7.8 eV for water). However, further evidence is required to prove the influence of bandgap energy of the medium on the filament length and diameter.

**Figure 5:**
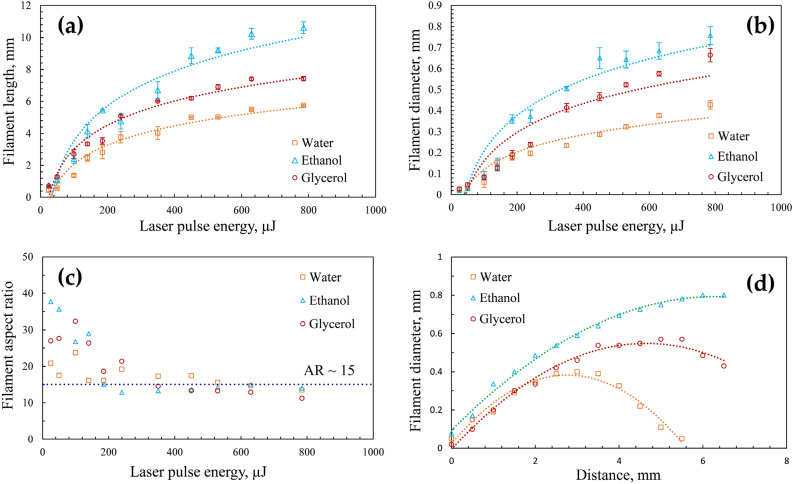
Variation of (**a**) filament bundle length, (**b**) filament bundle diameter, and (**c**) filament aspect ratio with laser pulse energy for different liquids. (**d**) Variation of filament diameter with distance along the optical axis for a laser energy of 785 µJ. The dotted lines in (**a**,**b**) represent logarithmic fit. The dotted line in (**c**) indicates the aspect ratio value of 15. The dotted lines in (**d**) represent a second-order polynomial fit.

The filament length and diameter have a logarithmic dependence on the pulse energy for all the liquids (Fig. 5a,b). The dotted lines represent the logarithmic fitting of the data. When the laser energy is low (< 200 µJ), the variation in the aspect ratio of the filament (length/diameter) with laser energy is stochastic. However, for comparatively high laser energies (> 200 µJ), the aspect ratio remains nearly constant at a value of ~ 15 (Fig. 5c). At the point of self-focus, the filament diameter (in the plane perpendicular to the camera) is small. The diameter increases with distance (along the optical axis) and reaches a maximum value near the center of the filament. The diameter subsequently decreases, reaching a minimum at the end of the filament. In the case of water, the filament diameter is symmetric with respect to the distance along the optical axis (Fig. 5d). The trend is similar for glycerol and ethanol; however, it would be entirely symmetric if the laser energy was raised beyond 785 µJ.

### Bubble size distribution

**Figure 6:**
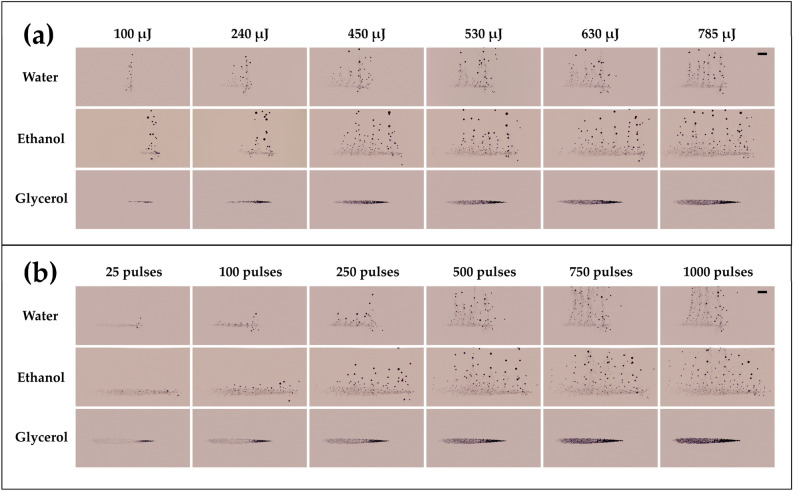
(**a**) Micro-bubble generation for different laser energies and liquid media (water, ethanol, and glycerol) for 500 pulses. (**b**) Micro-bubble generation as a function of the number of laser pulses for different liquid media (water, ethanol, and glycerol) at 785 µJ. The laser pulse comes from the left. The scale bar represents 1 mm.

The size distribution and population density of the persisting bubbles can be controlled by varying the laser pulse energy and the number of pulses for water and ethanol. For glycerol, however, due to its significantly higher viscosity (950 cP) compared to water (0.89 cP) and ethanol (1.1 cP), the bubbles agglomerate and remain stagnant at the location of their creation. For both water and ethanol, when the laser energy is low, the microbubbles are usually monodispersed (~ 20 µm) with a relatively low population density (Fig. 6a). The microbubbles tend to grow in size with increased laser energy and exhibit a polydisperse size distribution with diameters ranging from 10 µm to 250 µm.

The microbubbles have been found to be predominantly of two sizes, with a substantial population of small bubbles (~ 20 µm) and a few large bubbles (> 40 µm). The average size of the bubbles generated in ethanol (40 µm) is relatively larger than that in water (20 µm). It is important to note that there is a possibility of the presence of nanometer-sized bubbles, which are significantly smaller than the spatial resolution of the current experiments (~ 10 μm/pixel). However, the microbubbles generated with a size < 10 μm tend to die out rapidly as they shrink and then collapse since the pressure force of the smaller micro-bubbles is smaller and can be suppressed by the surrounding fluid pressure.

**Figure 7:**
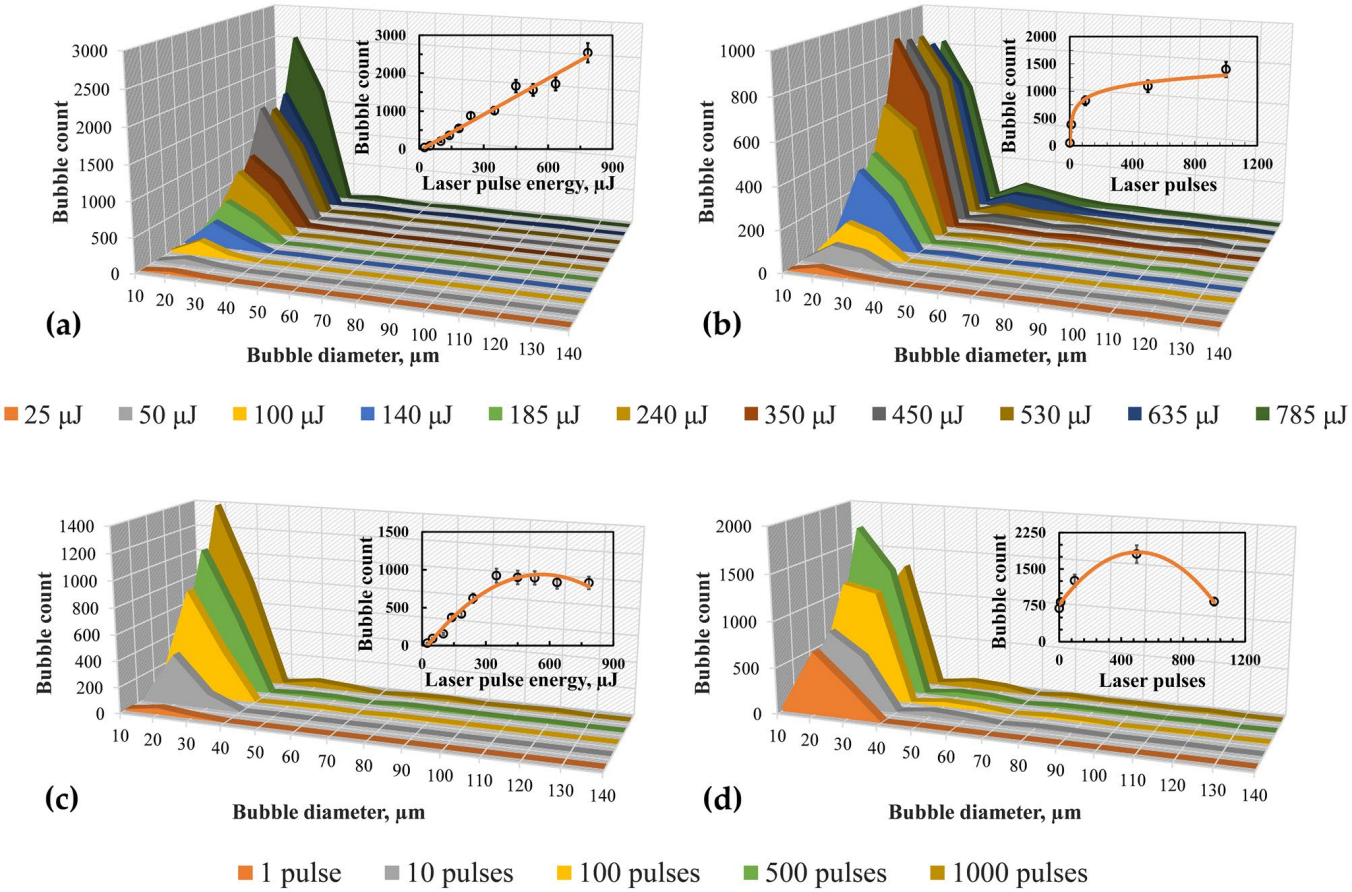
Size distribution of bubbles as a function of laser pulse energy for (**a**) water and (**b**) ethanol. Size distribution of microbubbles as a function of the number of laser pulses for (**c**) water and (**d**) ethanol at 785 µJ. Insets in (**a**) and (**b**) show the variation of bubble count (20 µm) with laser energy for water and ethanol, respectively. Insets in (**c**) and (**d**) show the variation of bubble count (20 µm) with laser pulses for water and ethanol, respectively. Solid lines in (**a**) and (**c**) indicate linear and logarithmic fit corresponding to water. Solid lines in (**b**) and (**d**) indicate second-order polynomial fit corresponding to ethanol.

As the number of pulses is increased, the population density and size of bubbles increase (see Fig. 6b). When the number of laser pulses is low, the bubble size distribution is nearly monodispersed (~ 20 µm). In contrast, a high number of pulses leads to the polydisperse size distribution. Figure 7a,b show the variation of microbubble count as a function of bubble diameter for different laser pulse energies for water and ethanol, respectively. Irrespective of the laser energy or the number of pulses, most bubbles generated are in the range of 10–40 µm for both liquids, with a peak at 20 µm.

In the case of water, with an increase in laser energy, the population density of the bubbles consistently rises (Fig. 7a). For a particular bubble size (20 µm), the bubble count increases linearly with the laser energy, as seen in the inset of Fig. 7a, where the solid line represents a linear fit. In the case of ethanol, however, the maximum bubble count reaches a peak value at 350 µJ, whereafter, the population begins to decline with a further augmentation of laser energy. The reduction in the bubble population beyond specific laser energy can be attributed to the coalescence of bubbles resulting in larger bubble sizes (> 40 µm), which is evident in Fig. 7b. The higher tendency of coalescence and hence the creation of larger bubbles in the case of ethanol can be associated with the generation of comparatively larger bubbles, especially near the focal region (will be discussed in section “Results and discussion”). The variation of bubble count for 20 µm size with respect to laser energy is depicted in the inset of Fig. 7b, where the solid line is a second-order polynomial fit.

A comparable trend of bubble size distribution is observed when the number of laser pulses is increased to a maximum of 1000 pulses for specific laser energy. For water, the bubble count rises with a gradual increase in the number of pulses (Fig. 7c). In particular, for 20 µm sized bubbles, the population increases logarithmically with an increase in the number of pulses (see inset of Fig. 7c). However, the bubble count reaches a maximum at 500 pulses for ethanol. With a further increase in the number of pulses, the bubble count reduces due to the coalescence of microbubbles (Fig. 7d). The solid line in the inset of Fig. 7d corresponds to the second-order polynomial fit.

### Bubble dynamics in the focal region

A single bubble grows and collapses near the focal region for all the laser energies (1 µJ to 785 µJ), which can be attributed to the optical breakdown process. Self-focusing in the liquid medium can lead to such high intensities that optical breakdown occurs at the geometrical focus^49,50^. A filamentation process (discussed in section “Results and discussion”) is usually followed by an optical breakdown near the geometrical focus. The expansion of this single bubble is primarily governed by the phase change from non-equilibrium plasma to gas-phase due to photo-ionization of the liquid. The influence of surface tension, viscosity, and the gas diffusion from the liquid into the bubble is negligibly small and can be disregarded^51–53^.

The growth, collapse, and consequent rebound of a single bubble are evaluated only for ethanol and water since the bubbles formed in glycerol are significantly closer to each other, eventually resulting in aggregation. This agglomeration makes it challenging to ascertain the growth and collapse of an individual bubble in the case of glycerol.

**Figure 8:**
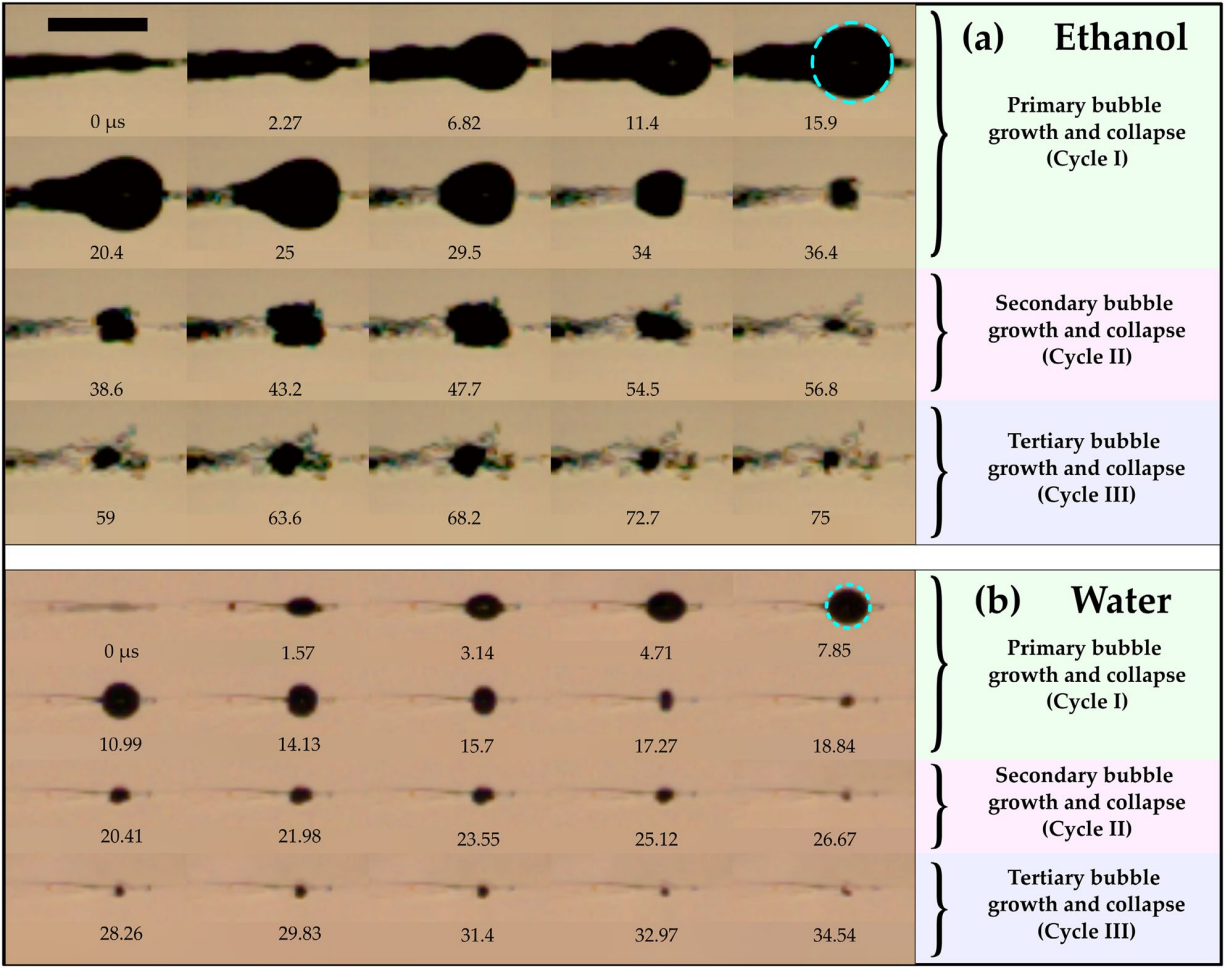
Growth and collapse of a single bubble near the geometrical focus in (**a**) ethanol and (**b**) water for 785 µJ, indicating growth and collapse cycles of primary, secondary, and tertiary bubbles. The laser pulse comes from the left. The scale bar indicates 1 mm.

The evolution of a single bubble at the geometrical focus for ethanol and water is shown in Fig. 8. At the focal region, the bubble starts to grow, and after attaining the maximal size, it begins to collapse (Fig. 8a,b) since the pressure inside the micro-bubble is significantly smaller than the external fluid pressure. This bubble further expands, followed by another collapse event, as seen in the successive images of bubble evolution (Fig. 8). These bubble growth and collapse cycles ("after-bounces") continue to occur multiple times (~ 5 cycles), which has been well modeled by Rayleigh-Plesset dynamics of free radial oscillations^54^. The bubble cycles viz., primary bubble (first cycle), secondary bubble (second cycle), and tertiary bubble (third cycle) for water and ethanol are depicted in Fig. 8a,b. It can be shown that the maximum bubble radius (${R}_{max}$) is interrelated to the collapse time (${T}_{coll}$), which is the time from bubble maximum to the ensuing minimum for a bubble collapsing in a liquid medium. The collapse time is given by^54^.

5 $${T}_{coll}=0.915{R}_{max}\sqrt{\frac{{\rho }_{liq}}{{P}_{stat}}},$$

where ${R}_{max}$ is the maximum bubble radius, ${\rho }_{liq}$ is the density of the liquid, and ${P}_{stat}$ is the external pressure. Equation 5 assumes that the growth and collapse phases of the bubble oscillation are symmetric. For the primary bubble, the Rayleigh collapse time (${T}_{coll}$) agrees well with the experimental bubble collapse time (${T}_{coll,exp}$) for both water (for 785 µJ, ${T}_{coll}$ = 8 µs and ${T}_{coll,exp}$ ~ 11 µs) and ethanol (for 785 µJ, ${T}_{coll}$= 15.7 µs and ${T}_{coll,exp}$~ 20 µs).

The lifetime of the bubble is higher for the first cycle, and it reduces with successive cycles for all the laser energies. For ethanol, at maximum laser energy (785 µJ), the lifetime of the primary, secondary, and tertiary bubbles is ~ 36, 20, and 18 µs, respectively. Similarly, for water (at 785 µJ), the lifetimes of the primary, secondary, and tertiary bubbles are ~ 18, 7, and 6 µs, respectively.

A significant difference in the bubble sizes pertaining to distinct cycles is found for water and ethanol. For ethanol, the maximum size of the primary, secondary, and tertiary bubbles is ~ 388, 248, and 196 µm, respectively. For water, on the other hand, the maximum size of the primary, secondary, and tertiary bubbles is ~ 176, 96, and 77 µm, respectively. It is noticed that, in the case of ethanol, the main bubble (highlighted with a dashed circle) overlaps with several other bubbles (Fig. 8a) compared to a single isolated bubble for water (Fig. 8b). With time, these bubbles merge and collapse as a single bubble (34 µs in Fig. 8a).

**Figure 9:**
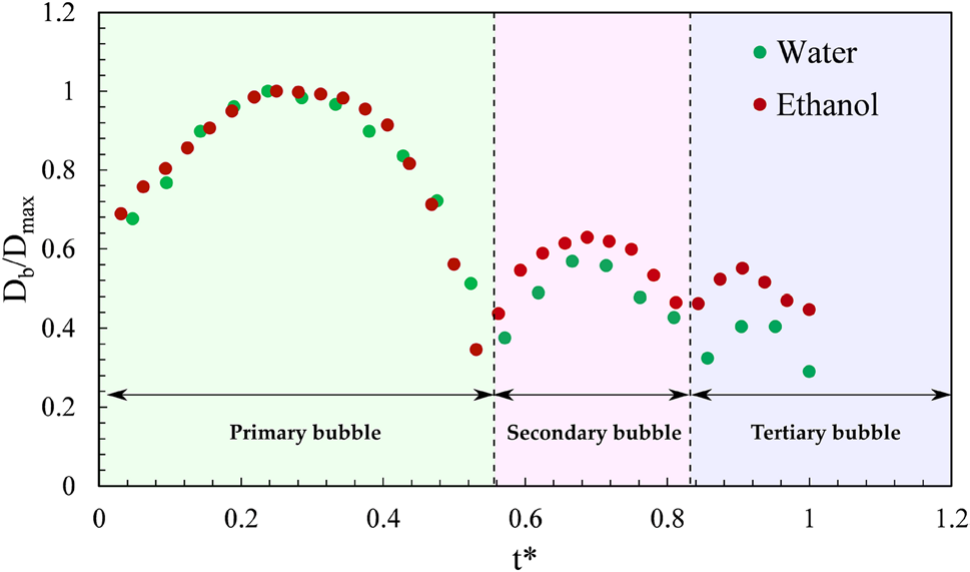
The growth and collapse cycles of the single bubble formed near the focal region for a laser energy of 785 µJ. The instantaneous bubble diameter (D_b_) is normalized with maximum bubble diameter (D_max_) and t* denotes instantaneous time normalized with the total bubble cycle time.

The temporal evolution of bubble diameter (D_b_/D_max_) with normalized time (t*) of the primary, secondary, and tertiary bubbles for water and ethanol is presented in Fig. 9. Here, t* represents instantaneous time normalized with the total bubble cycle time. The variation of the normalized bubble diameter of water overlaps with that of ethanol.

**Figure 10:**
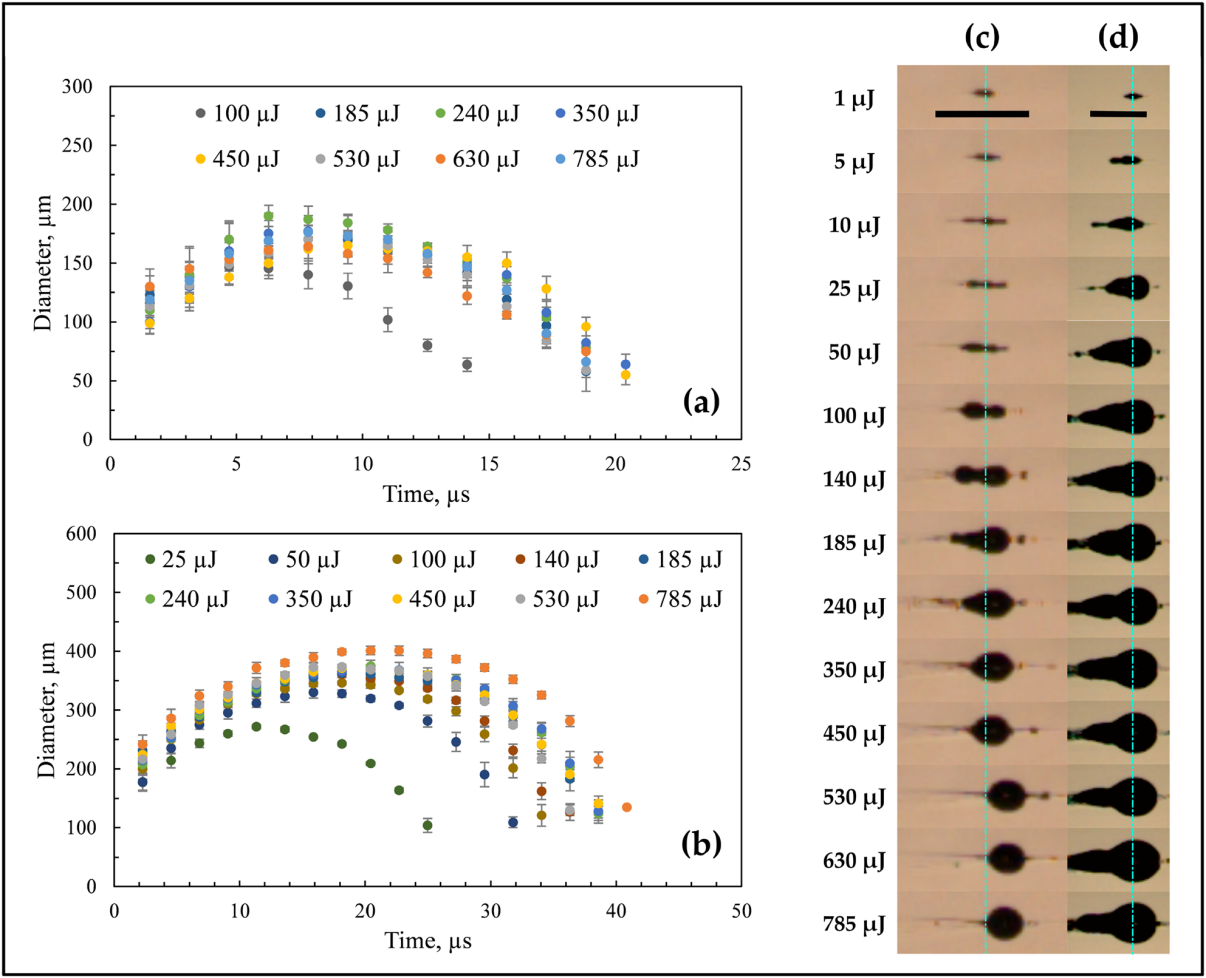
The growth and collapse of a single bubble for different laser energies for (**a**) water and (**b**) ethanol. The shape and size of a single bubble for different laser energies for (**c**) water and (**d**) ethanol. The dotted lines represent the center of the focal region. The laser pulse comes from the left. The scale bars indicate 1 mm.

The first oscillation cycle of the bubbles for water and ethanol at different laser energy is shown in Fig. 10a,b, respectively. For ethanol, the maximum bubble diameter increases with the increase in the laser pulse energy. The maximum bubble diameters are clearly distinguishable for ethanol, whereas the difference is not discernible for water. This can be attributed to the shape changes in the case of water (see Fig. 10c), especially for lower laser energies (< 240 µJ).

The single bubble at its maximum diameter for different laser pulse energies is shown in Fig. 10c,d. It is observed that the size and shape of the single bubble can be controlled effectively by varying the laser pulse energy for different liquids. At specific laser energies, the size and shape of the bubbles are distinguishable for different liquids (see Fig. 10c,d). In the case of water, the bubble shape evolves from a cylindrical configuration at low laser energy (< 50 µJ) to a spherical shape at the highest laser energy (785 µJ). In the case of ethanol, the shape of the bubble remains the same at laser energies > 25 µJ. Likewise, the bubble size remains constant at high laser energies (> 185 µJ). Unlike water, where only a single isolated bubble grows at high laser energies, multiple bubbles are witnessed to overlap and interact with each other and grow simultaneously for ethanol at all the laser pulse energies (see Fig. 10a,b). The aggregation of bubbles occurs since the bubble formation is not only confined at the focal spot but instead distributed over the Rayleigh length of the laser beam along the direction of the incident laser. Especially for high laser pulse energies, when several closely spaced bubbles are formed near the focal spot, the coalescence of multiple bubbles becomes evident (see Fig. 10d).

The energy of the bubble (*E*_*B*_) is proportional to the cube of its maximum radius and is given by^55^.

6 $${E}_{B}=\frac{4\pi {p}_{0}}{3}{R}_{max}^{3}.$$

The energy of the bubble can also be expressed in terms of the laser pulse energy (*E*_*L*_), giving

7 $${E}_{B}=\eta {E}_{L} .$$

**Figure 11:**
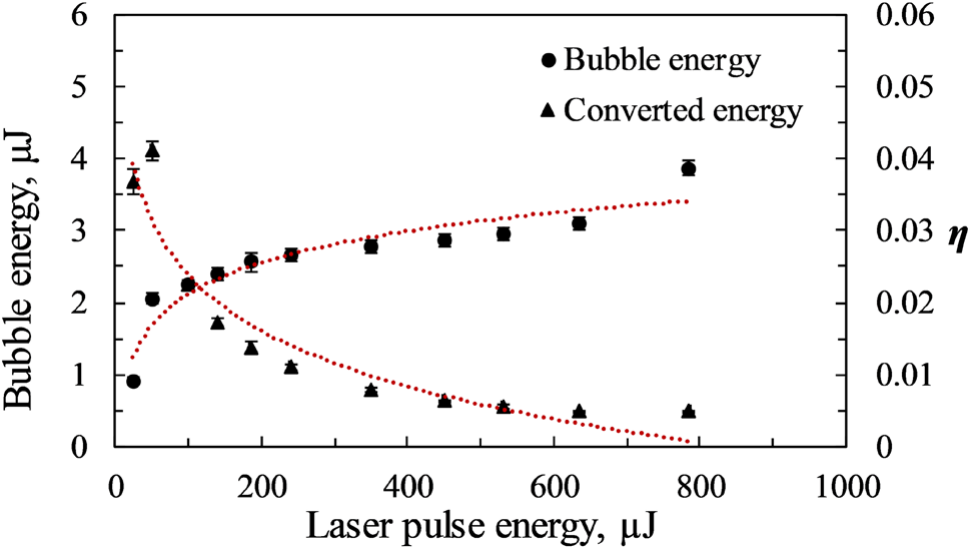
Variation of bubble energy and share of the pulse energy (*η*) for different incident laser energies.

Here, *η* is the share of the laser pulse energy converted to the bubble's energy. Figure 11 shows the logarithmic increase of bubble energy with the increase in the incident laser pulse energy. As expected, the energy converted (*η*) from the incident laser pulse to the bubble energy decreases as the laser pulse energy increases.

**Figure 12:**
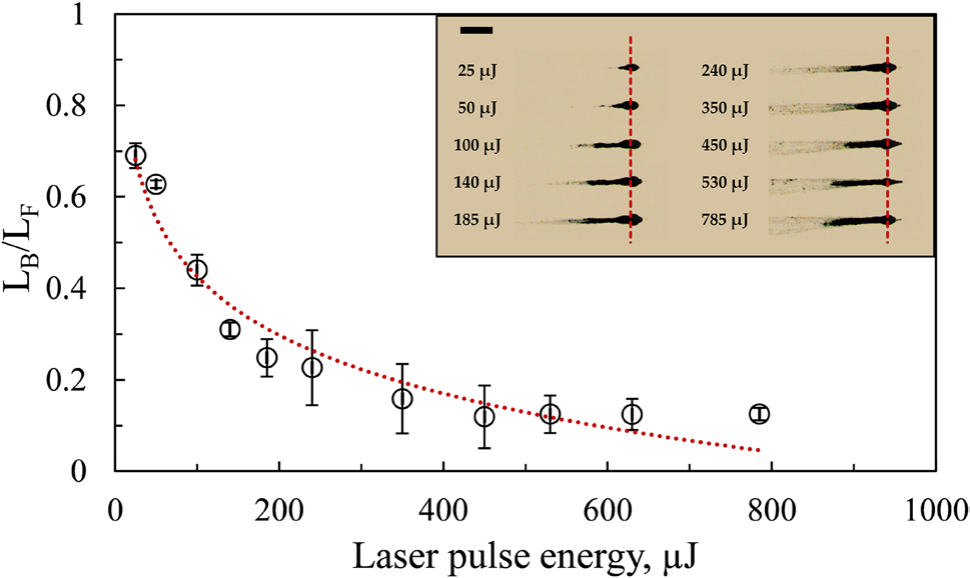
The dependence of the ratio of agglomerated bubble length (L_B_) at the focal region to the total filament length (L_F_) on the laser pulse energy in ethanol. Inset shows the experimental images of the agglomerated bubbles in the filament. The dotted lines in the inset represent the focal region. The scale bar indicates 500 µm.

Figure 12 shows the dependence of the ratio of agglomerated bubble length (L_B_) near the focal region to the filament length (L_F_) on laser energy for ethanol. The inset in Fig. 12 displays the bubble formation at the focal spot with a single laser pulse for different laser energies. When the laser energy is low (< 25 µJ), the bubbles generated are smaller in size with a relatively short length (~ 200 µm). As the energy increases, multiple bubbles occur, leading to agglomeration. This agglomeration increases as the laser energy increases (Fig. 12). The ratio of agglomerated bubble length to the filament length reduces logarithmically with laser energy, indicating the dominance of self-focusing at a high laser energy. This dominance is visible in the form of the filament tail extending towards the direction of the focusing lens (see inset of Fig. 12).

**Figure 13:**
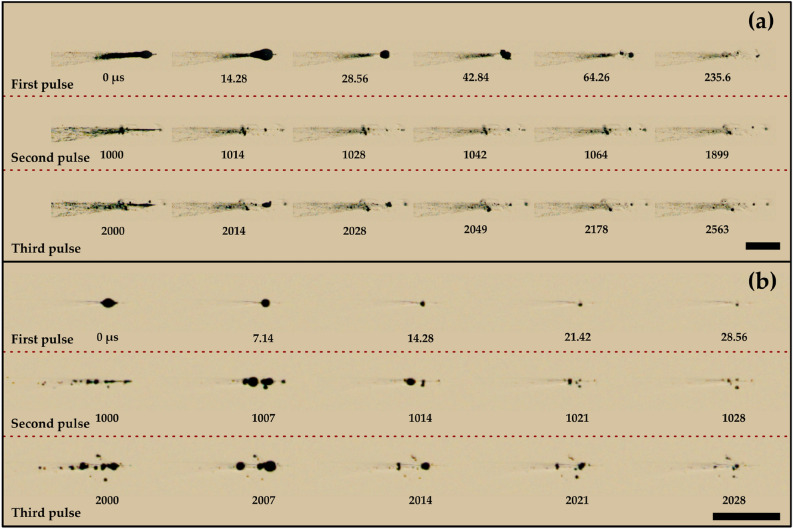
Influence of multiple laser pulses on the bubble dynamics near the focal region in (**a**) ethanol and (**b**) water for the pulse energy of 785 µJ. The scale bars indicate 1 mm.

The agglomeration phenomenon is significantly affected when multiple pulses are introduced into the liquid pool. The influence of multiple pulses in ethanol and water on the dynamics of bubbles at the focal region is shown in Fig. 13a,b, respectively. As discussed before, introducing a single pulse in a liquid pool results in the formation of a single bubble at the focal region, which collapses into smaller bubble fragments. However, when a second pulse is introduced, it interacts with the persistent bubble fragments from the first pulse and does not allow any agglomeration and growth of the clustered bubbles. A similar sequence of events is seen for succeeding laser pulses (see the introduction of the third pulse in Fig. 13a). Nevertheless, the multiple laser pulses result in more bubbles since the persistent bubble fragments from the previous laser-liquid interaction become the site for the formation of new bubbles. Therefore, the population of the bubbles grows with more pulses (also described in section “Results and discussion”). It is important to note that the change in temperature due to the introduction of multiple laser pulses can be considered negligible since it has been shown that, in the case of femtosecond pulses, the heat-affected zone is minimal^56,57^.

### Bubble merging and coalescence

**Figure 14:**
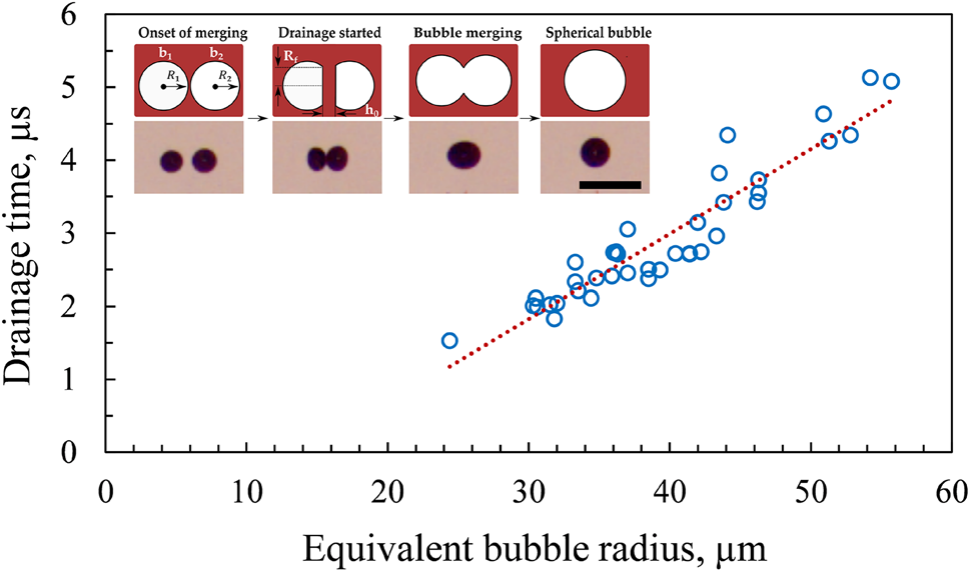
Dependence of drainage time on the equivalent bubble radius in water for 785 µJ laser energy. The inset shows the sequence of events involved in the bubble merging process resulting from the drainage of the thin liquid film. The time step between two consecutive images in the inset is 0.3 ms. The scale bars indicate 500 µm.

When laser pulses are introduced into the liquid medium, bubble coalescence occurs in different filament regions. As expected, this coalescence phenomenon is enhanced when multiple consequent laser pulses are introduced. The coalescence of two adjoining microbubbles primarily occurs in three phases. At first, the two bubbles come in contact, forming an initial thin film between them. Thereafter, the liquid film contracts and becomes a delicate layer which ruptures, leading to a rapid coalescence of the bubbles. Cain and Lee^58^ have reported a similar sequence of events for acoustic bubbles. It was noted that the bubble coalescence mainly depends on the approach velocity, fluid viscosity, and the force between the microbubbles. The radius of the approaching bubbles can be approximated to an equivalent bubble radius, ${R}_{eq}$ which can be defined as^59^.

8 $$\frac{2}{{R}_{\mathrm{eq}}}=\frac{1}{{\mathrm{R}}_{1}}+\frac{1}{{\mathrm{R}}_{2}},$$

where ${\mathrm{R}}_{1}$ and ${\mathrm{R}}_{2}$ are the radii of the two approaching bubbles. The equivalent bubble radius obtained in this work ranges from 30 to 55 µm. During coalescence, a thin liquid film forms between the contact region of the two bubbles. The thickness of the film grows further and eventually reaches a critical thickness. At this stage, the thin liquid film between the bubbles ruptures and the bubbles fuse to form a single larger bubble (see the inset of Fig. 14). This coalescence time or film drainage time can be calculated using the expression of Kirkpatrick and Lockett^60^.

9 $${t}_{Ds}={R}_{f}\sqrt{\frac{{\rho }_{liq}{R}_{eq}}{16\upsigma }ln\frac{{h}_{0}}{{h}_{c}}},$$

where ${R}_{f}$ is the radius of the bubble contacting area, ${h}_{0}$ is the initial liquid film thickness, and ${h}_{c}$ is the critical film thickness at which the film ruptures. Oolman and Blanch^61^ have reported this film's initial and critical thickness to be in the range of 1–10 µm and 0.01 µm, respectively. We have considered the value for ${h}_{0}$ as 5 µm and ${h}_{c}$ as 0.01 µm. The density (${\rho }_{liq}$) and surface tension (σ) of water used to calculate drainage time is 998 kg/m^3^ and 0.072 N/m, respectively.

The drainage time increases linearly with the equivalent bubble radius (Fig. 14). The inset depicts a schematic and experimental observation of the important events in the bubble merging process. The coalescence of two bubbles, b_1_ and b_2_, having radii R_1_ and R_2_, begins as they approach each other. The drainage initiates as they come closer at a distance of ${h}_{0}$, which is essentially the initial thickness of the thin film. This interaction results in the flattening of the interposed film between the bubbles, which subsequently drains to a critical thickness (${h}_{\mathrm{c}}$). The radius of the thin film (contact area) is indicated as ${R}_{\mathrm{f}}$ which eventually ruptures, followed by the coalescence, resulting in the formation of a single bubble (see inset of Fig. 14). The drainage time is of the order of a few microseconds (~ 2 to 5 µs).

The coalescence or rebound of the approaching bubbles is influenced by the induction of an acoustic field inside the liquid by the laser pulse. When bubbles are exposed to such an acoustic field, the process of coalescence is further complicated by the Bjerknes forces exerted on the micro-bubbles. Moreover, the introduction of the laser pulse causes the bubbles to oscillate/pulsate. This bubble pulsation is a manifestation of the acoustic field.

Depending on the size of the bubbles, the coalescence can occur due to both primary and secondary Bjerknes forces, with the latter being more dominant at close ranges (~ 1 mm)^62^. In this work, we primarily measure the secondary Bjerknes force, which causes the bubble-to-bubble interaction. The secondary Bjerknes force (${\mathrm{F}}_{\mathbf{B}}$) is described by Doinikov as^63^.

10 $${\text{F}}_{{\mathbf{B}}} = \frac{{4{{\uppi }}\rho }}{{{\text{r}}^{2} _{{12}} }} < {\text{R}}_{1} ^{2} {\dot{\mathrm{R}}}_{1} {\text{R}}_{2} ^{2} {\dot{\mathrm{R}}}_{2} > ,$$

where ${\mathrm{r}}_{12}$ is the separation distance between the two micro-bubbles taken from the centre of the bubbles, ${\mathrm{R}}_{1}$ and ${\mathrm{R}}_{2}$ are the radii of the two approaching bubbles, and ${\dot{{R}}}_{1}$ and ${\dot{{R}}}_{2}$ are the radial velocities of the respective bubbles. Equation 10 indicates that the volume change in one acoustic cycle is related to both the change in radii of the two bubbles and their radial velocities.

**Figure 15:**
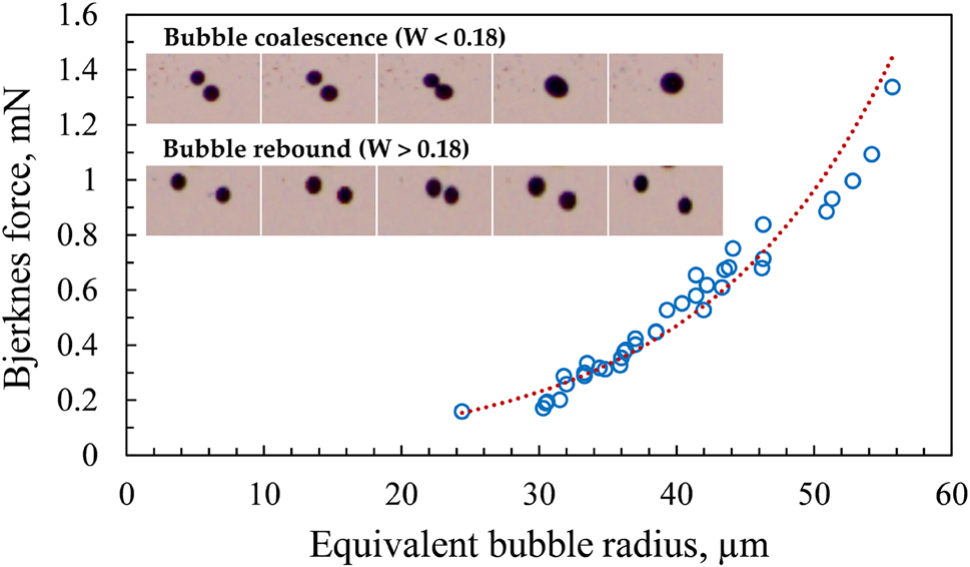
Variation of Bjerknes force with equivalent bubble radius for water at 785 µJ. The inset shows the two cases of bubble interaction based on the value of the Weber number. The time step between two consecutive images in the inset is 0.3 ms.

The variation of Bjerknes force as a function of equivalent bubble radius for water is depicted in Fig. 15. It is evident that the Bjerknes force exponentially increases with the equivalent bubble radius. The Bjerknes force between the bubbles ranges from ~ 0.15 to 1.35 mN. We witnessed that bubbles bounce off each other in some instances while others coalesce when a new pulse is introduced in the liquid pool.

This coalescence or rebound of the approaching micro-bubbles can be predicted using a dimensionless number known as the Weber number (W), which is given by.

11 $${\text{W}} = {{\rho _{{liq}} U^{2} R_{{{\text{eq}}}} } \mathord{\left/ {\vphantom {{\rho _{{liq}} U^{2} R_{{{\text{eq}}}} } \sigma }} \right. \kern-\nulldelimiterspace} \sigma },$$

where ${\rho }_{liq}$ is the density of the liquid, *U* is the velocity of the two approaching bubbles, $\sigma$ is the surface tension and ${R}_{eq}$ is the equivalent radius of the bubbles. The value of this parameter signifies whether the approaching bubbles will coalesce or rebound. If W < 0.18, the two bubbles tend to coalesce, whereas when W > 0.18, the bubbles experience rebound owing to high approach velocity (see inset of Fig. 15). In the current study, W is predominantly < 0.18, indicating that the coalescence process dominates over the rebound of bubbles. The coalescence or rebound via Bjerknes force is witnessed primarily near the geometrical focus, indicating a high magnitude of acoustic force. In addition, bubbles with an equivalent radius of 30 to 40 µm tend to coalesce more frequently due to their significantly larger population (see section “Results and discussion”). It has also been noticed that the magnitude of Bjerknes force is unaffected by the laser pulse energy and liquid media.

## Conclusions

This work delineates the distinct attributes of femtosecond laser-induced filamentation and accompanying bubble dynamics in liquid media. We have revealed the spatio-temporal evolution and interaction of the filamentation-induced bubbles as a function of laser pulse energies (∼1 to 800 µJ) and the number of laser pulses (up to 1000 pulses) in different liquid media (water, ethanol, and glycerol). The filament length and diameter are found to have a logarithmic dependence on the laser energy, irrespective of the medium. It is observed that ethanol has the maximum filament length and diameter, followed by glycerol and water, due to its low bandgap energy. The size distribution of the persisting micro-bubbles is controlled by varying the laser pulse energy and the number of pulses. Irrespective of the laser energy or the number of pulses, most bubbles generated are in the range of 10–40 µm. For lower laser energy and the number of pulses, the bubbles generated are monodisperse in nature (~ 20 µm). As the laser energy and number of pulses are increased, the bubble population and the polydispersity of the bubbles increases. A significant difference in the single bubble size and shape between water and ethanol is observed in the focal region.

The results also reveal that the introduction of consecutive pulses leads to strong interaction and coalescence of the pulsating bubbles via Bjerknes force (~ 0.2 to 1.5 mN) due to the laser-induced acoustic field generation. The present work also reveals that large bubbles cannot be generated by filamentation in liquids at kHz repetition rate due to the interaction of the previously formed bubbles with the laser pulse. Therefore, high repetition rate lasers are more suitable for generating small bubbles. This technique of microbubble generation using a femtosecond laser allows precise control and prediction of the size, location, and polydispersity of the generated bubbles, making them useful in microfluidic applications.

## Data Availability

The datasets used and/or analysed during the current study are available from the corresponding author on reasonable request.
