# Author Correction: Controlling bubble generation by femtosecond laser-induced filamentation

**DOI:** 10.1038/s41598-022-23194-w

**Published:** 2022-11-04

**Authors:** D. Chaitanya Kumar Rao, Veena S. Mooss, Yogeshwar Nath Mishra, Dag Hanstorp

**Affiliations:** 1 Department of Physics, University of Gothenburg, 41296 Gothenburg, Sweden; 2 NASA-Jet Propulsion Laboratory, California Institute of Technology, Pasadena, CA 91109 USA; 3 Present Address: Department of Aerospace Engineering, Indian Institute of Technology Kanpur, Kanpur, 208016 India

Correction to:* Scientific Reports* 10.1038/s41598-022-20066-1, published online 21 September 2022

The original version of this Article contained errors.

In Figure 7, the insets in panels (c) and (d) were omitted.

Consequently, the Figure 7 legend was incomplete. The legend now reads:

“**Figure 7**. Size distribution of bubbles as a function of laser pulse energy for (**a**) water and (**b**) ethanol. Size distribution of microbubbles as a function of the number of laser pulses for (**c**) water and (**d**) ethanol at 785 µJ. Insets in (**a**) and (**b**) show the variation of bubble count (20 µm) with laser energy for water and ethanol, respectively. Insets in (**c**) and (**d**) show the variation of bubble count (20 µm) with laser pulses for water and ethanol, respectively. Solid lines in (**a**) and (**c**) indicate linear and logarithmic fit corresponding to water. Solid lines in (**b**) and (**d**) indicate second-order polynomial fit corresponding to ethanol.”

**Figure 7:**
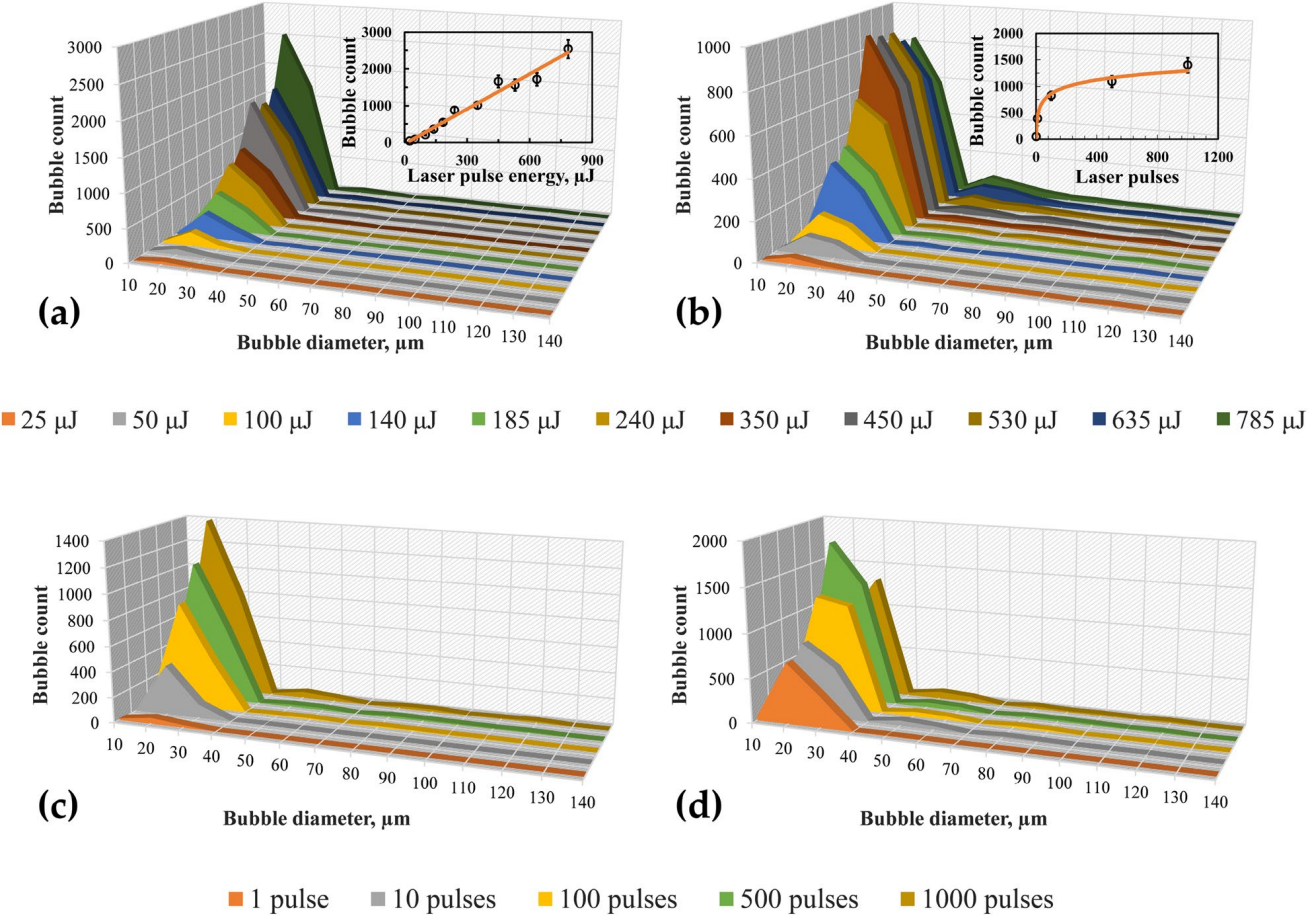
Size distribution of bubbles as a function of laser pulse energy for (**a**) water and (**b**) ethanol. Size distribution of microbubbles as a function of the number of laser pulses for (**c**) water and (**d**) ethanol at 785 µJ. Insets in (**a**) and (**b**) show the variation of bubble count (20 µm) with laser energy for water and ethanol, respectively. Solid lines in (**a**) and (**c**) indicate linear and logarithmic fit corresponding to water. Solid lines in (**b**) and (**d**) indicate second-order polynomial fit corresponding to ethanol.

The original Figure 7 and accompanying legend appear below.

The original Article has been corrected.

